# Biomechanical assessment of the rheumatoid foot

**DOI:** 10.1186/1757-1146-5-S1-I4

**Published:** 2012-04-10

**Authors:** Keith Rome, Gordon Hendry

**Affiliations:** 1 Health & Rehabilitation Research Institute, AUT University, Auckland, 0627, New Zealand; 2 University of Western Sydney, Sydney, Australia

## Background

The aim of the workshop is to conduct a biomechanical foot assessment of the rheumatoid foot. Participants will be involved in an interactive workshop that includes patients with rheumatoid arthritis. We will examine foot-related outcome tools designed to evaluate foot function, pain and disability. Delegates will be divided into small groups to analyse and interpret temporal and spatial gait parameters and plantar pressure measurements.

Upon completion of this workshop, participants should be able to:

• Describe key components of a foot and ankle examination for the rheumatology patient

• Discuss how current technology pertains to conceptualizing and treating patients with rheumatic disease

• Describe the clinical and research applications of validated foot outcome assessment instruments

• Formulate evidence-based non-pharmacological treatment strategies based on pain mechanisms, unique clinical features and biomechanical characteristics

• Demonstrate how biomechanical concepts and related treatment strategies can be used to address complex cases of rheumatological patients with foot pain, impairments and disability

